# Downregulation of microRNA‐124‐3p promotes subventricular zone neural stem cell activation by enhancing the function of BDNF downstream pathways after traumatic brain injury in adult rats

**DOI:** 10.1111/cns.13845

**Published:** 2022-04-28

**Authors:** En‐Ming Kang, Yi‐Bin Jia, Jia‐You Wang, Guan‐Yi Wang, Hui‐Jun Chen, Xiao‐Yan Chen, Yu‐Qin Ye, Xin Zhang, Xin‐Hong Su, Jing‐Yu Wang, Xiao‐Sheng He

**Affiliations:** ^1^ Department of Neurosurgery, Xijing Hospital Airforce Military Medical University (Fourth Military Medical University) Xi'an China; ^2^ Department of Neurosurgery PLA 163rd Hospital (Second Affiliated Hospital of Hunan Normal University) Changsha China; ^3^ Teaching and Research Support Center Engineering University of Chinese Armed Police Force Xi'an, Shaanxi China

**Keywords:** bioinformatics, brain‐derived neurotrophic factor (BNDF), miR‐124, neural stem cells (NSCs), subventricular zone (SVZ), traumatic brain injury (TBI)

## Abstract

**Aims:**

In this study, the effect of intracerebral ventricle injection with a miR‐124‐3p agomir or antagomir on prognosis and on subventricular zone (SVZ) neural stem cells (NSCs) in adult rats with moderate traumatic brain injury (TBI) was investigated.

**Methods:**

Model rats with moderate controlled cortical impact (CCI) were established and verified as described previously. The dynamic changes in miR‐124‐3p and the status of NSCs in the SVZ were analyzed. To evaluate the effect of lateral ventricle injection with miR‐124‐3p analogs and inhibitors after TBI, modified neurological severity scores (mNSSs) and rotarod tests were used to assess motor function prognosis. The variation in SVZ NSC marker expression was also explored. Kyoto Encyclopedia of Genes and Genomes (KEGG) pathway enrichment analysis of predicted miR‐124‐3p targets was performed to infer miR‐124‐3p functions, and miR‐124‐3p effects on pivotal predicted targets were further explored.

**Results:**

Administration of miR‐124 inhibitors enhanced SVZ NSC proliferation and improved the motor function of TBI rats. Functional analysis of miR‐124 targets revealed high correlations between miR‐124 and neurotrophin signaling pathways, especially the TrkB downstream pathway. PI3K, Akt3, and Ras were found to be crucial miR‐124 targets and to be involved in most predicted functional pathways. Interference with miR‐124 expression in the lateral ventricle affected the PI3K/Akt3 and Ras pathways in the SVZ, and miR‐124 inhibitors intensified the potency of brain‐derived neurotrophic factor (BDNF) in SVZ NSC proliferation after TBI.

**Conclusion:**

Disrupting miR‐124 expression through lateral ventricle injection has beneficial effects on neuroregeneration and TBI prognosis. Moreover, the combined use of BDNF and miR‐124 inhibitors might lead to better outcomes in TBI than BDNF treatment alone.

## Graphical abstract

Administration of miR‐124 inhibitors enhances SVZ NSCs activation in TBI rats. Such process is partly achieved by up‐regulating BDNF downstream PI3K/Akt3 and Ras signaling pathways.

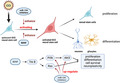

## 1 INTRODUCTION

Traumatic brain injury (TBI) is a leading cause of injury‐related death and disability worldwide and has a devastating impact on patients and their families.^1^ TBI is characterized by a heterogeneous group of pathologies initiated by diverse mechanisms and disruptions to various neurological functions, such as executive actions, cognitive ability, attention, memory data processing, and language abilities.^2^ For years, updates to treatment guidelines based on strong evidence for TBI treatment have been slow, and many multicenter clinical trials of medical interventions that have promising preclinical results not led to significant differences in neurological outcomes.^1^, ^3^

The adult mammalian brain is capable of structural and functional repair, plasticity, and regeneration. Adult neural stem cells (NSCs) residing in the subventricular zone (SVZ) produce DCX^+^ neuroblasts that migrate along the rostral migratory stream and differentiate into neurons.^4^ NSCs need to exit the quiescence state to undergo neurogenesis and differentiation. This process is controlled *in vivo* by comprehensive signaling processes involving, for example, local cell–cell interactions, secreted factors in the blood or cerebrospinal fluid, and long‐range signaling triggered by neuronal inputs or factors secreted from the choroid plexus in the SVZ niche.^5^ After brain injury, the environment changes in the SVZ stem cell niche; for example, vasculature permeability is increased, inducing NSCs to migrate to the injured cortex and differentiate into protective astrocytes.^6^ Additionally, DCX^+^ neuroblasts continuously migrate to the corpus callosum 2–4 days after brain injury.^7^ Transcriptome analysis based on single‐cell RNA sequencing (RNA‐seq) has revealed an increase in the proportion of “primed” quiescent and active NSCs in the SVZ 2 days post‐ischemia. Both primed and active NSCs drive the expression of genes related to protein synthesis and the cell cycle.^8^ These findings have highlighted reactive SVZ NSCs conferred protection after TBI, and therefore, promoting SVZ NSC activation might provide beneficial outcomes for patients.

MicroRNAs (miRNAs) are single‐stranded noncoding RNAs 18–25 (~22) nucleotides in length that play important roles in the post‐transcriptional regulation of gene expression.^9^ To date, 2588 mature miRNAs processed from 1881 precursor miRNAs have been annotated in the human genome,^10^ and most of these miRNAs are highly conserved across species.^11^ miRNAs regulate many target genes through both perfect and imperfect base pairing of the miRNA seed region to the 3′ untranslated region (UTR) of mRNA, making it difficult to gain a complete understanding of the complex miRNA function.^12^ MiR‐124 is one of the most abundant miRNAs expressed in the adult brain and has been found to be involved in both prenatal and postnatal neuronal differentiation.^13^ MiR‐124 regulates Sox9 to impair NSC proliferation and facilitate NSC differentiation.^14^ In the case of cerebral ischemia, the level of miR‐124 is reduced in the SVZ, and this reduction inhibits neuronal progenitor cell proliferation because jagged‐1 (JAG1) expression is repressed.^15^ An *in vitro* study of oxygen–glucose deprivation/re‐oxygenation (OGD/R) showed that a miR‐124 inhibitor promoted growth‐associated protein‐43 (GAP‐43) expression and axonal growth.^16^ Although the function of miR‐124 in cerebral ischemia and other central nervous system diseases has been previously and extensively explored,^17^ the role that miR‐124 plays in SVZ NSCs *in vivo* after TBI remains unclear.

In the present study, we used an *in vivo* controlled cortical impact (CCI) rat model to explore the function of miR‐124 in adult SVZ NSCs after TBI. We found that lateral cerebral ventricle injection with miR‐124 inhibitors enhanced SVZ NSC proliferation and improved motor function in adult TBI rats. Based on the results of a Kyoto Encyclopedia of Genes and Genomes (KEGG) pathway analysis on predicted miR‐124 targets, we verified that this regulation pattern was partially implemented and affected the function of pathways downstream of BDNF, such as the PI3K/Akt3 and Ras signaling pathways.

## 2 MATERIALS AND METHODS

### 2.1 Controlled cortical impact (CCI) model

Animal experiments were authorized by the Animal Care and Use Committee of Air Force Military Medical University, Xi'an, China. Healthy adult male SD rats weighing 300–350 g were purchased from the Center of Experimental Animals of the Air Force Military Medical University. Rats were raised in the facility with a 12‐h day and night cycle and with stable temperature and humidity. All rats were allowed to eat and drink freely and had adequate space for free activity. A CCI device (Hatteras Instruments Inc., Cary, NC, USA) was used to establish the TBI model. After anesthetization with 10% chloral hydrate (intraperitoneal injection), the rats were placed on a brain stereotaxis instrument (Kopf Instruments, Tujunga, CA, USA). The rat head was wiped with 70% ethanol, and the scalp was cut at the midline. A bone harvest drill was used to make a hole 2.5 mm in diameter, 2.0 mm posterior to the bregma, and 2.0 mm lateral to the sagittal suture on the right side of the skull. The CCI device was then used to induce a moderate TBI model with a general parameter (impact depth, 3 mm; impact velocity, 5.0 m/s; dwell time, 100 ms).^18^ After the injury, the scalp incision was closed, and the rats were placed on a constant temperature plate to maintain their core temperature. The research strategy used in this study complied with the instructions of the Animal Research Reporting *in vivo* Experiment (ARRIVE) 2.0 guidelines.^19^

### 2.2 Administration of drugs

Rats were anesthetized with 10% chloral hydrate (intraperitoneal injection) and placed in a stereotaxic frame. Stereotaxic injections were made with a Hamilton syringe using the following coordinates: 1.5 mm rostral to bregma, 1 mm lateral to midline, and 5.5 mm ventral to the skull surface.^20^ Rats in the control or sham group were given 10 μL of phosphate‐buffered saline (PBS, pH 7.4); rats in the negative control (NC), agomir, or antagomir groups were given gradient RNA doses of 100, 200, and 400 pmol, and the efficacy of these concentrations was evaluated 3 days after injection. The sequences of the agomir and antagomir were as follows: agomir‐124‐3p 5′ to 3′: sense UAA GGC ACG CGG UGA AUG CC, antisense GGC AUU CAC CGC GUG CCU UA; antagomir‐124‐3p 5′ to 3′: GGC AUU CAC CGC GUG CCU UA. For TBI rats, administration treatment was given on two days after TBI induction; at this time, the rats were injected with 400 pmol NC, agomir, or antagomir RNA. For the BDNF groups, 10 μg of BDNF (Catalog no. 248‐BDB, R&D Systems, Minneapolis, MN, USA) was diluted in 10 μl RNase‐free PBS (pH 7.4) combined with NC, agomir, or antagomir RNA and administered by bilateral intraventricular injection to each rat. After injection, the needle was left in place for 10 min before being slowly withdrawn. The mortality rate of the rats after surgery was 1/7, and no difference was found among the different treatment groups. The cause of death of rats that died 24 h after surgery was ascribed to anesthesia or stress, and these rats were excluded from the functional analysis.

### 2.3 Neurobehavioral training and evaluation

The mNSS was obtained prior to TBI (or after sham surgery) and at 1, 3, 5, 7, 14, and 30 days after TBI (or sham) surgery. Briefly, the test included a composite of tasks that the rats completed for the assessment of the motor, sensory, reflex, and balance ability postinjury. Rats with an abnormal score (score > 0) before surgery were excluded from the experiment.^21^ The higher the score was, the more serious the defect was considered.

A rotarod test was performed as described herein.^22^, ^23^ Briefly, rats were placed on a rotating drum with a speed slowly increasing from 0 to 40 rpm within 5 min; that is, the speed increased 2 rpm every 10 s for 200 s. When the speed was held constant at 40 rpm, the duration (seconds) that the rat stayed on the rod was recorded; that is, the time between the initiation of the test and the time at which the animal fell off the drum was recorded. Animals were tested three times per day for 3 days before surgery.

### 2.4 Tissue preparation

Rats with only CCI were sacrificed 1, 3, 5, and 7 days after the operation. The rats in the injection group were sacrificed 7 days after CCI induction. Rats were deeply anesthetized and transcardially perfused with 0.1 M PBS (pH 7.4) before the brains were collected. The SVZ tissue for real‐time quantitative PCR and Western blotting was taken by microdissection from tissue approximately 1 mm adjacent to the lateral corner of the lateral ventricle. For immunofluorescence (IF), rats were perfused transcardially with 0.1 M PBS (pH 7.4) followed by a fixative solution containing 4% paraformaldehyde in PBS (pH 7.4) for 2 h. The brain was removed and fixed in the same fixative solution at 4°C for an additional 2 h.^24^

### 2.5 Immunostaining

Brain sections (4 μm thick) were deparaffinized with xylene, rehydrated with ethanol, and then subjected to antigen retrieval with citric acid and sodium citrate antigen retrieval solution (pH = 6.0) at 120°C for 100 s. The sections were then successively incubated in hydrogen peroxide for 20 min, 0.3% Triton X‐100 in PBS for 10 min, and donkey serum for 45 min to block nonspecific signals at room temperature. Then, the primary antibodies were incubated at 4°C for 20 h.^24^ The samples were then incubated with secondary antibodies for 4 h at room temperature. The secondary antibodies were Alexa Flour™ 488 donkey anti‐mouse IgG (H+L) (1:500, Catalog no. A21202; Thermo Fisher Scientific, USA) and Alexa Flour™ 594 donkey anti‐rabbit IgG (H+L) (1:1000, Catalog no. A21207; Thermo Fisher Scientific).

Nuclei were counterstained with 4′,6‐diamidino‐2‐phenylindole (DAPI) at room temperature for 5 min. After being washed five times with PBS, the sections were mounted with antifade mounting medium (Beyotime, P0131, Shanghai, China) and covered. All experimental procedures were necessarily performed to minimize the exposure of the tissues to light. The primary antibodies are listed in Table 1.

**TABLE 1:** Primary antibodies used in IF and WB

Antibodies	Source	Catalog	RRID	Dilution	Usage
GFAP	Abcam	ab33922	AB_732571	1:1000	IF
Nestin	Abcam	ab11306	AB_1640723	1:500	IF
DCX	Abcam	ab254133	N/A	1:500	IF
Ki67	Abcam	ab16667	AB_302459	1:500	IF
DCX	Abcam	ab254133	N/A	1:1000	WB
BDNF	Abcam	ab205067	N/A	1:2000	WB
PI3KCA	Abcam	ab183957	N/A	1:1000	WB
Phospho‐Akt1/2/3	Abcam	ab192623	AB_2269641	1:1000	WB
NT‐3	Abcam	ab263864	AB_2884942	1:1000	WB
Akt3	CST	#14982	AB_2716311	1:1000	WB
GAPDH	CST	#5174	AB_10622025	1:1000	WB
Ras	CST	#3339	AB_2269641	1:1000	WB
Phospho‐MEK1/2	CST	#9154	AB_2138017	1:1000	WB
Phospho‐p44/42 MAK	CST	#4370	AB_2315112	1:1000	WB
Phospho‐PI3K p85/p55	Affinity	AF3242	AB_2834668	1:1000	WB

Abbreviations: N/A, not available; CST, cell signaling technology; IF, immunofluorescence; WB, Western blotting.

### 2.6 Cell counting

Ki67‐positive, DCX‐positive, and double‐labeled cells in the SVZ along the lateral walls of the lateral ventricles (beginning 1.18 mm anterior to the bregma) were counted by an observer blinded to the experimental conditions. Five to seven 6‐μm paraffin coronal sections per animal (*n* = 6 per group)^25^ spaced 120 μm apart were assessed with an upright fluorescence microscope (OLYMPUS, BX53, Shinjuku, Tokyo, Japan) with a 40x objective. A CellSens Standard system (OLYMPUS) was used to record z‐stack images and thus confirm the colocalization of GFAP‐ and Nestin‐labeled cells in the SVZ. The results are expressed as the average number of GFAP‐ and Nestin‐positive cells, Ki67‐positive cells, and DCX‐positive cells in the SVZ.

### 2.7 qRT–PCR assay

Total RNA was isolated from SVZ tissue using TRIzol reagent (Invitrogen). miRNAs were reverse transcribed with a PrimeScript RT reagent kit (TaKaRa, Tokyo, Japan). qRT‐PCRs were performed in triplicate to detect the expression of miR‐124‐3p with SYBR Premix Ex Taq II (Takara) using a CFX96 Real‐Time PCR Detection System (Bio‐Rad, Hercules, CA, USA). The relative quantification of miR‐124‐3p was performed by the 2^−ΔΔCt^ method, and U6 was employed as an endogenous control to normalize the miR‐124‐3p data. The primer sequences were as follows: miR‐124‐3p F: 5′‐GCT TAA GGC ACG CGG‐3′; miR‐124‐3p R: 5′‐GTG CAG GGT CCG AGG‐3′; U6 F: 5′‐CTC GCT TCG GCA GCA CAT A‐3′; U6 R: 5′‐CGC TTC ACG AAT TTG CGT G‐3′; miR‐124‐3p RT: 5′‐GTC GTA TCC AGT GCA GGG TCC GAG GTA TTC GCA CTG GAT ACG ACG GCA TTC‐3′; and U6 RT: 5′‐CGC TTC ACG AAT TTG CGT GTC AT‐3′.

### 2.8 Western blotting

The total protein in the SVZ tissue was extracted, and a BCA protein kit (Beyotime) was used to quantify the protein concentrations. Ten micrograms of protein extract mixed with 5 × SDS–PAGE sample loading buffer (Solarbio) were separated by SDS–PAGE and electrotransferred onto polyvinylidene fluoride (PVDF) membranes (Millipore, Billerica, MA, USA). The membranes were blocked with 5% skimmed milk or BSA fraction V (for blocking phosphorylated proteins) (Beyotime, Shanghai, China) powder solution for 2 h, followed by incubation with primary antibodies overnight at 4°C. Next, the blots were probed with horseradish peroxidase (HRP)‐conjugated secondary antibody (1:1000, Catalog no. #7074; Cell Signaling Technology, USA) for 1 h. The protein bands were evaluated with an enhanced chemiluminescence (ECL) kit (BeyoECL Plus; Beyotime) with GAPDH used as the internal reference. The primary antibodies are listed in Table 1.

### 2.9 Statistical analysis

All of the data are expressed as the means ± SD. GraphPad Prism 7.0 software was used to perform Student's t‐test or two‐way ANOVA followed by Tukey's multiple comparison test. Nonparametric tests (Jonckheere–Terpstra test and Kruskal–Wallis test) were performed with data that were not normally distributed. *p* < 0.05 was considered to be significant.

## 3 RESULTS

### 3.1 Time course of miR‐124‐3p and SVZ NSCs post‐TBI

**FIGURE 1:**
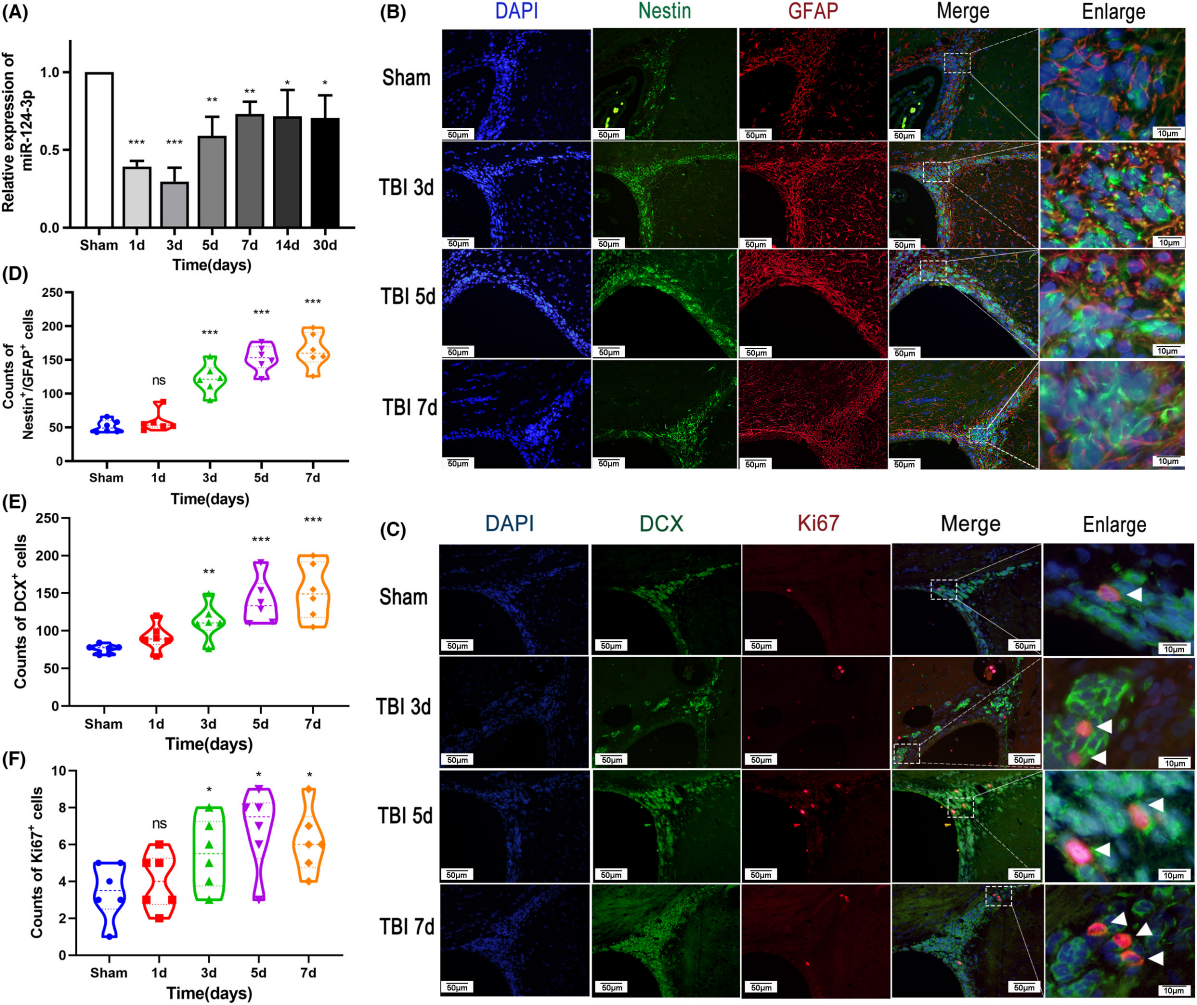
Time course of miR‐124‐3p expression and the status of NSCs in the SVZ post‐TBI. (A) Qrt‐PCR analysis of the expression of miR‐124‐3p. The expression of miR‐124‐3p decreased sharply in the first 3 days after TBI and then increased slightly and was sustained until Day 30. The expression level at Day 30 was still lower than that in the sham group. (B–F) The status of NSCs was examined with immunofluorescence was performed 3, 5, and 7 days after TBI (*n* = 6 per group). (E) Representative photographs showing double immunofluorescence staining for Nestin (green) and GFAP (red) in the SVZ postinjury. Scale bar = 100 μm. (B) The number of Nestin^+^/GFAP^+^ cells increased gradually post‐TBI. (F) Representative photographs showing double IF staining for DCX (green) and Ki67 (red) in the SVZ postinjury. Scale bar = 100 μm. (C) The number of Ki67^+^ cells increased post‐TBI. (D) DCX^+^ cells increased post‐TBI. The data are presented as the means ± SD. ^⁎^ *p* < 0.05, ^⁎⁎^ *p* < 0.01, and ^⁎⁎⁎^ *p* < 0.001 vs. the sham group

The expression of miR‐124‐3p in the SVZ was investigated 1, 3, 5, 7, 14, and 30 days post‐TBI (*n* = 3 per group), and qRT–PCR showed a downregulation of miR‐124‐3p. The expression of miR‐124‐3p decreased dramatically in the first 3 days post‐TBI, and then, the expression level increased slightly but remained lower than the physiological level and was sustained at this level until day 30 (Figure 1A). The status of the NSCs in the SVZ was also explored by immunostaining 3, 5, and 7 days post‐TBI (*n* = 6 per group) (Figure 1B,C). The results showed that the number of GFAP^+^/Nestin^+^ stem cells and DCX^+^ progenitor cells increased gradually after TBI (Figure 1D,E). The number of Ki67^+^ dividing cells increased and reached a peak on day 5 (Figure 1F). These data suggested that miR‐124‐3p was downregulated and that NSCs were activated in the SVZ of adult rats in response to TBI.

### 3.2 MiR‐124‐3p increased neurological deficits after TBI

**FIGURE 2:**
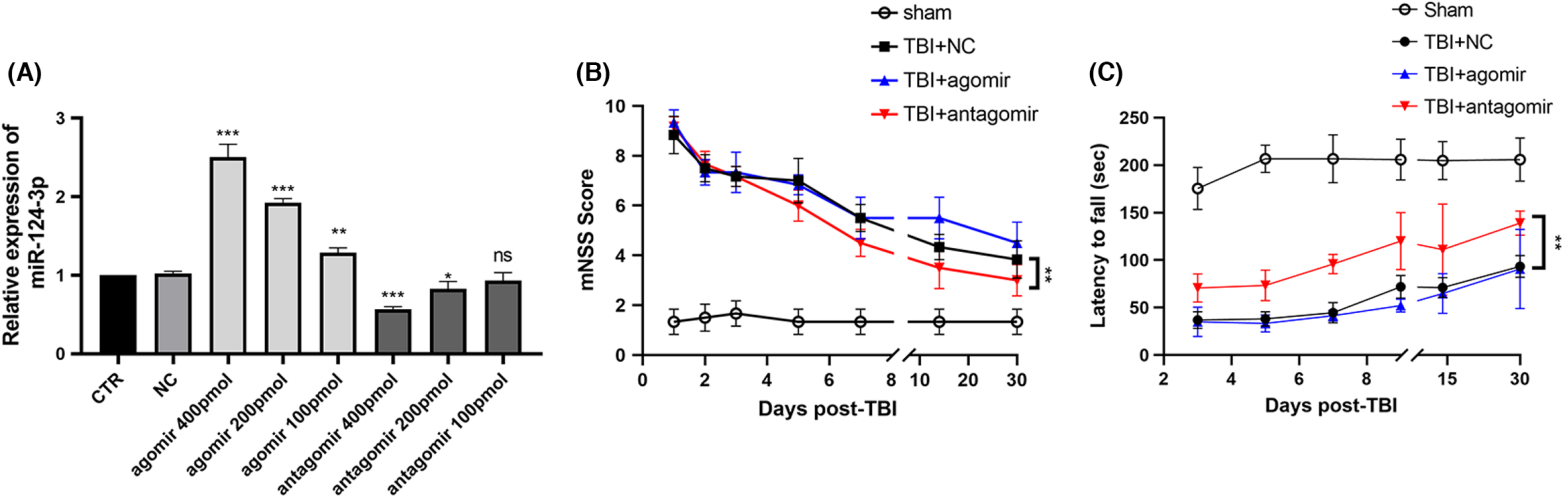
MiR‐124‐3p interference efficiency and behavioral evaluation. (A) Agomir‐124‐3p, antagomir‐124‐3p, and NCs were injected into the lateral ventricle at a volume of 10 μl per side, and the SVZ tissue was dissected 3 days postinjection. At concentrations of 100, 200, and 400 pmol per side, the injected RNAs all led to significant changes in miR‐124‐3p expression, and 400 pmol RNAs was applied in the following experiments. ^⁎^ *p* < 0.05, ^⁎⁎^ *p* < 0.01, and ^⁎⁎⁎^ *p* < 0.001 vs. the NC group. CCI rats then achieved double‐sided lateral ventricle injection of NC, agomir or antagomir on the first day after injury and were then evaluated on the basis of mNSS scores (B) and a rotarod test (C). The results were analyzed by nonparametric test (mNSS) or ANOVA (rotarod), and the performance of the TBI + antagomir group was found to be better than that of the TBI + agomir or TBI + NC group. No significant difference was found between the TBI + agomir and TBI + NC groups. The data are presented as the means ± SD. ^⁎^ *p* < 0.05, ^⁎⁎^ *p* < 0.01, and ^⁎⁎⁎^ *p* < 0.001 vs. the TBI + NC group. *n* = 6/group

To confirm the role of miR‐124‐3p after TBI, we administered miR‐124‐3p to the CCI rats. As SVZ NSCs extend cilia through the ependymal rosettes to contact the cerebrospinal fluid in the ventricular space,^26^ we injected the lateral cerebral ventricle with miR‐124‐3p agomir or antagomir to explore their effects on TBI rats. qRT‐PCR was used to explore the expression of miR‐124 3 days after injection, and a dose of 400 pmol RNA was adopted as the appropriate intervention for the following experiments (Figure 2A). Rats (*n* = 6 per group) were evaluated by mNSS and rotarod test, and the results showed no difference between the groups before surgery. Following CCI and injection, the TBI rats exhibited worse mNSS or rotarod test performance than the sham rats (Figure 2B,C). By consecutively monitoring the performance of each group for 30 days, no difference was found in mNSS between the TBI + NC group and the TBI + agomir group, but the TBI + antagomir group obtained better outcomes (Figure 2B). In the rotarod test, the TBI + antagomir group achieved the lowest fall ratio on days 5, 7, and 14, and the TBI + agomir group had the highest fall ratio (Figure 2C). These results implied that downregulation of miR‐124‐3p correlated with a better motor function outcome in adult TBI rats.

### 3.3 MiR‐124‐3p attenuates SVZ NSC activation post‐TBI

**FIGURE 3:**
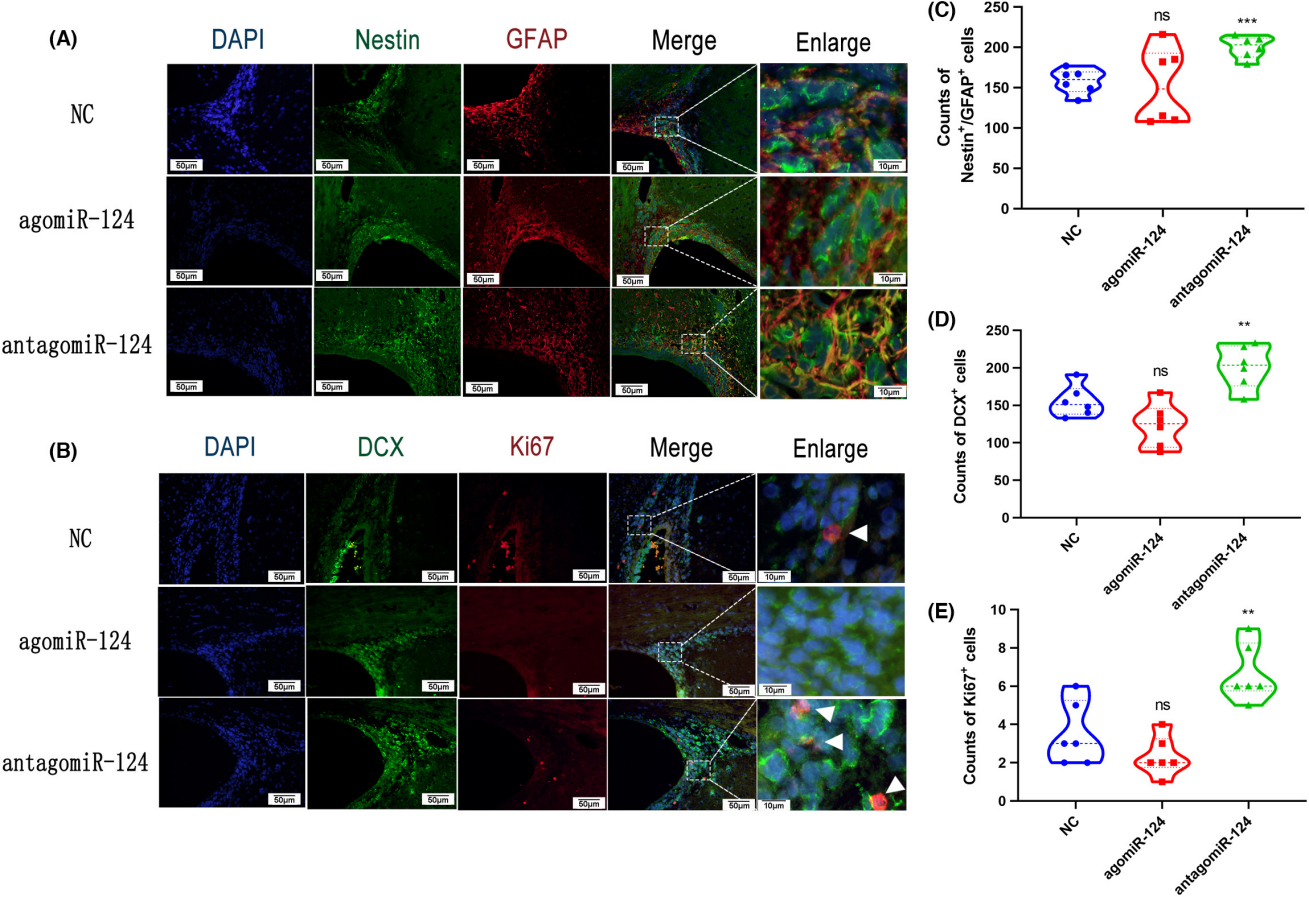
Immunofluorescence of NSCs in the SVZ after injection of agomiR‐124‐3p or antagomir‐124‐3p post‐TBI. The CCI rats received a double‐sided lateral ventricle injection of NC, agomiR, or antagomir 24 h after injury, and the rats were sacrificed 7 days after TBI induction. (A) Representative photographs showing double immunofluorescence staining for Nestin (green) and GFAP (red) in the SVZ. Scale bar = 100 μm. (B) Representative photographs showing double immunofluorescence staining for DCX (green) and Ki67 (red) in the SVZ. Scale bar = 100 μm. The numbers of Nestin^+^/GFAP^+^ cells (C), DCX^+^ cells (D), and Ki67^+^ cells (E) in the TBI + antagomir group were significantly higher than those in the NC group. The data are presented as the means ± SD. ^⁎^ *p* < 0.05 and ^⁎⁎^ *p* < 0.01 vs. the NC group

To explore whether miR‐124‐3p exerted an impact on SVZ NSCs after TBI, we performed IF assays to explore the variation in NSC markers in each group on Day 7 post‐TBI (*n* = 6 per group) (Figure 3A,B). Consistent with the behavioral outcomes described above, the TBI + antagomir group had the largest number of GFAP^+^/Nestin^+^ cells, DCX^+^ cells, and Ki67^+^ cells (Figure 3C–E). For the TBI + NC group and TBI + agomir group, no difference was found in GFAP^+^/Nestin^+^ cell numbers. The numbers of DCX^+^ cells, and Ki67^+^ cells in the TBI + agomir group were lower than those in the TBI + NC group. The IF assay results indicated that decreased miR‐124‐3p might play a role in promoting NSC activation in the SVZ after TBI.

### 3.4 Bioinformatics analysis of MiR‐124‐3p targets

**FIGURE 4:**
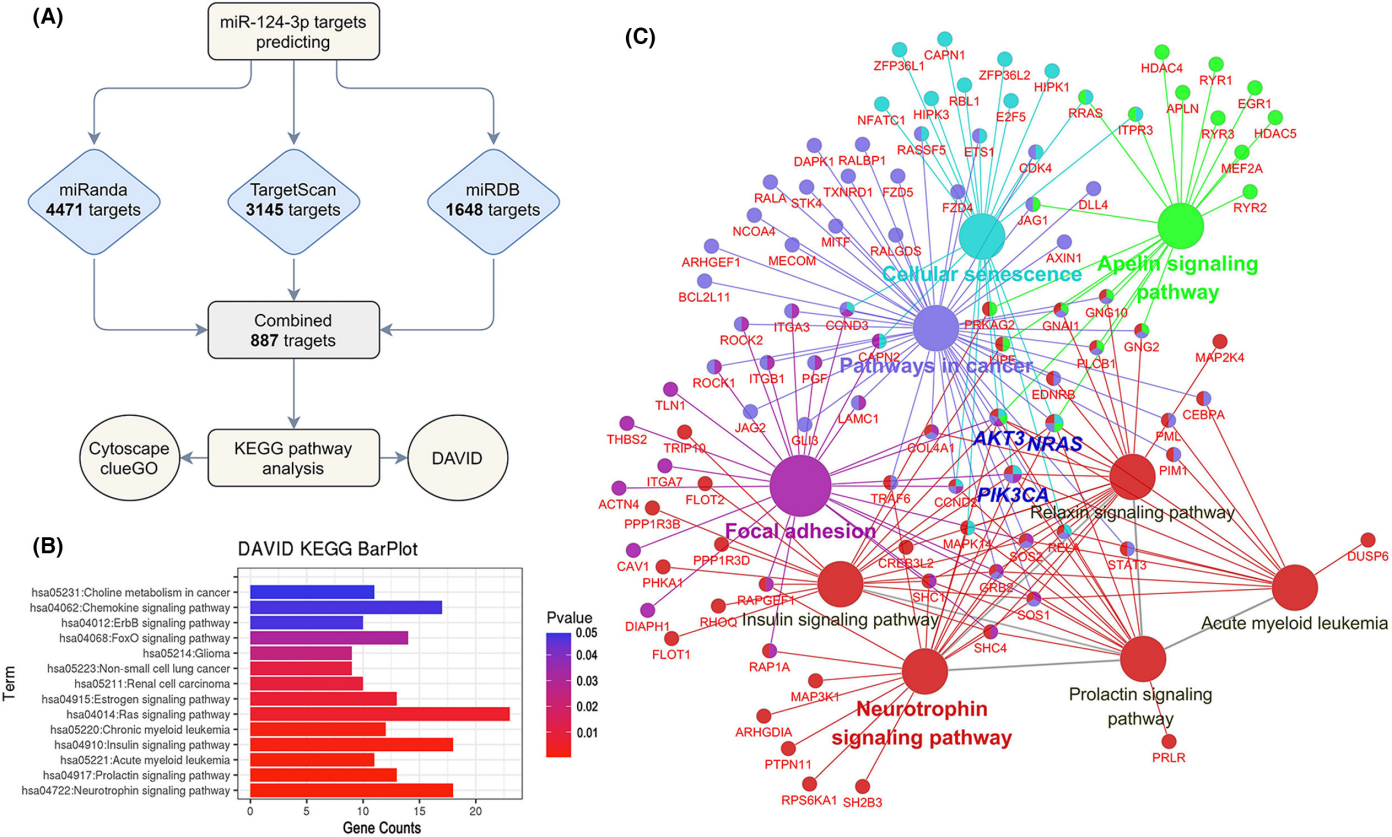
Targets and functional analysis of miR‐124‐3p. (A) Workflow showing miR‐124‐3p target screening and the tools used for the KEGG pathway analysis. (B) DAVID KEGG analysis results for the predicted targets. The results were ranked by P‐value. (C) Cytoscape ClueGO results based on the KEGG pathway analysis. Pathways with a *p*‐value < 0.05 are visualized, and the relative target genes and their correlation between each pathway are shown. Pathways with the same color represent the same group whose gene overlap ratio was > 80%

The biological function of miR‐124‐3p was predicted through a bioinformatics analysis, the workflow of which is schematically presented in Figure 4A. Target genes of miR‐124‐3p were screened using TargetScan (http://www.targetscan.org), miRDB (www.mirdb.org), and miRanda (www.miranda.org), and a total of 887 targets were screened for use in a pathway enrichment analysis. KEGG pathway analysis results based on the DAVID database (Figure 4B) and Cytoscape ClueGO (Figure 4C) showed that the predicted targets were mostly enriched in “neurotrophin signaling pathway,” “prolactin signaling pathway” and “acute myeloid leukemia.” Among the targets in these pathways, NRas, PIK3CA, and Akt3 were found to be participants in the most pathways. These results suggested that miR‐124‐3p broadly regulates these pathways and that NRas, PI3KCA, and Akt3 are the pivotal participants in these processes. MiR‐124‐3p targets participated in Neutrophin signaling pathways and prolectin pathways are highlighted in Supplementary Materials.

### 3.5 The MiR‐124‐3p antagomir promoted the expression of Akt3, PI3KCA, and Ras

**FIGURE 5:**
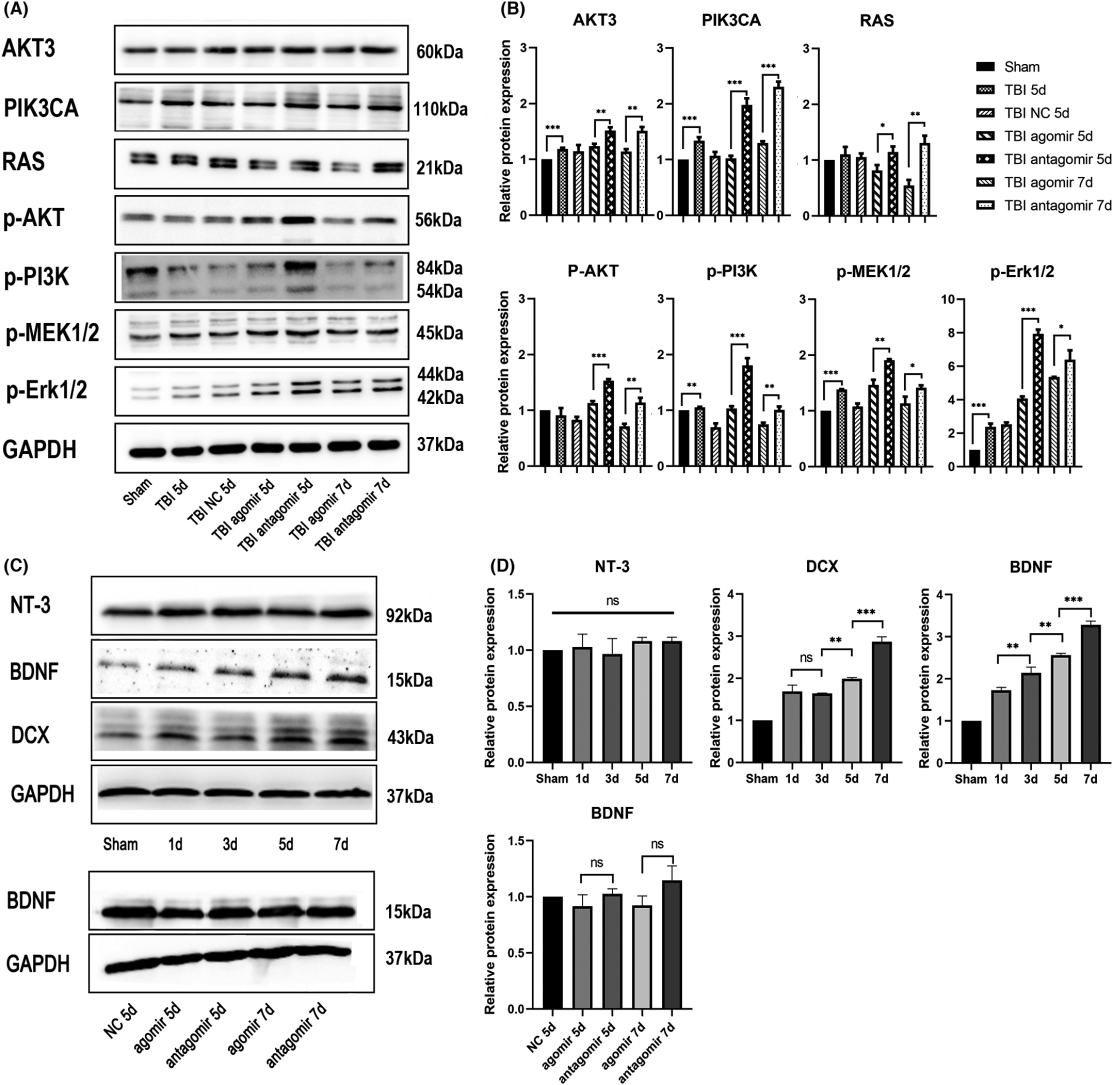
Western blotting of SVZ tissues post‐TBI. (A) MiR‐124‐3p affected the expression of neurotrophin signaling pathway molecules after TBI. Western blot (WB) analysis of Akt3, PIK3CA, Ras, phospho‐Akt, phospho‐PI3K, phospho‐MEK1/2, and phospho‐Erk1/2 expression in SVZ tissues of TBI rats injected with agomir‐124‐3p or antagomir‐124‐3p 5 and 7 days after injury. (B) Statistical analysis of the relative protein expression of each molecule. All proteins analyzed were found to be significantly differentially expressed between the agomir group and antagomir group. (C) The expression of NT‐3, BDNF, and DCX in the SVZ tissue was tested 1, 3, 5, and 7 days after TBI. (D) Statistical analysis of the relative protein expression of each molecule. All data are presented as the means ± SD. ^⁎^ *p* < 0.05. *n* = 3/group. Full length western blot scans for the cropped images are presented in Figure S1

We next detected the expression of Ras, PI3KCA, and Akt3 in all the experimental groups (*n* = 3 per group) 5 and 7 days after TBI (Figure 5A,B). Western blot analysis showed that the expression of all three proteins increased after TBI, which indicated their proliferation‐promoting effect after TBI. Additionally, the TBI + agomir group appeared to have lower levels of Akt3, PIK3CA, and Ras expression than the TBI + antagomir group, which implied that the expression of these genes was regulated by miR‐124‐3p. As the Ras, PI3K, and Akt are activated by phosphorylation, the expression of phospho‐PI3K, phospho‐MEK, and phospho‐Erk was also tested. Consistent with the results described above, the levels of phospho‐MEK and phospho‐Erk were significantly reduced in the TBI + agomir group compared with the TBI + antagomir group. These results confirmed that miR‐124‐3p inhibited the expression and function of Ras, PI3KCA, and Akt3, which subsequently inhibited the activation of the PI3K/Akt pathway and Ras pathway.

### 3.6 MiR‐124‐3p inhibited BDNF function in activating SVZ NSCs after TBI

**FIGURE 6:**
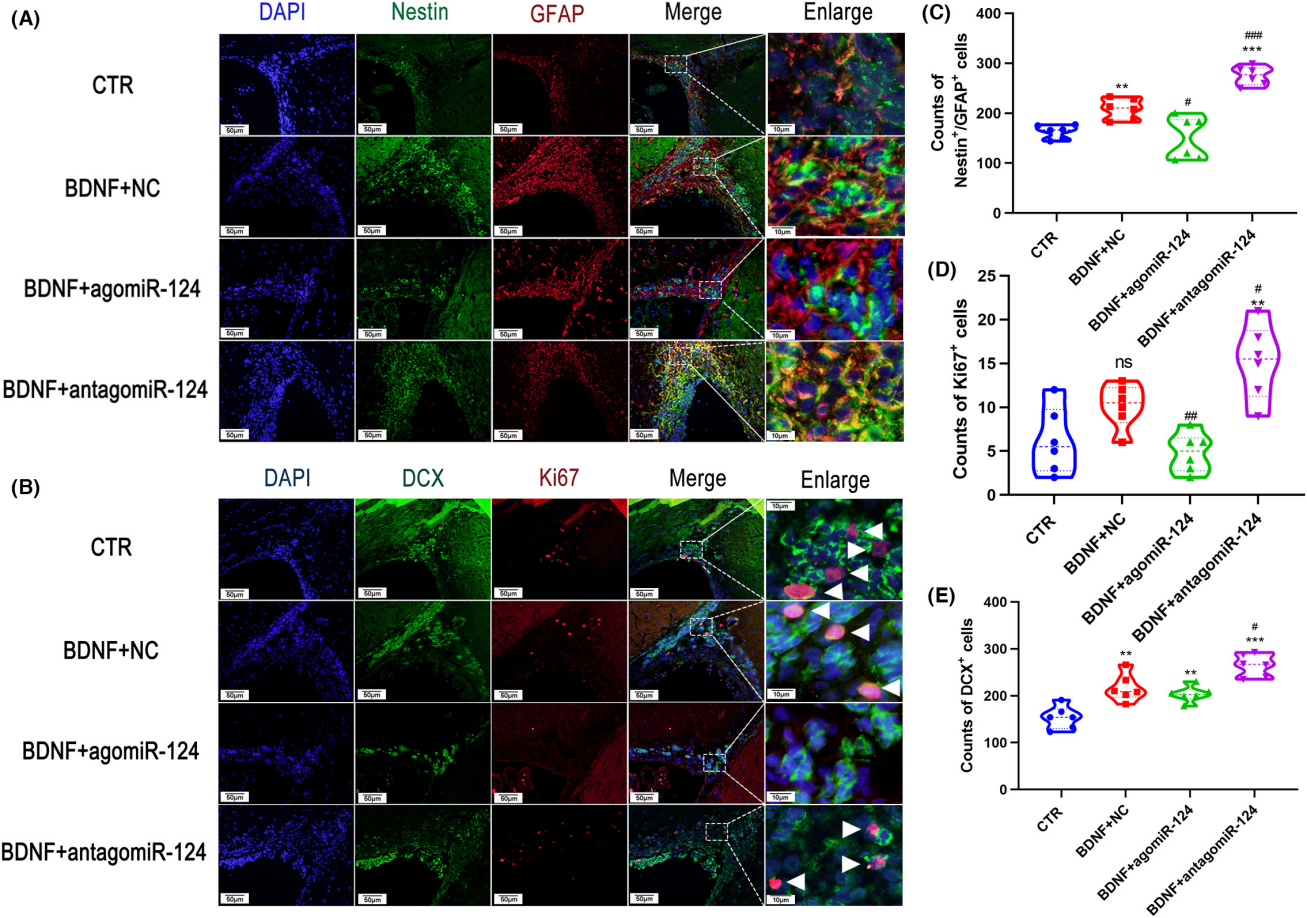
MiR‐124‐3p inhibited BDNF activation of SVZ NSCs after TBI. Immunofluorescence microphotographs of NSCs in the SVZ of CCI rats were taken after they were injected with BDNF, BDNF + agomiR or BDNF + antagomir. The rats were sacrificed 7 days after TBI induction, and Nestin^+^/GFAP^+^ cells (A), DCX^+^ cells, and Ki67^+^ cells (B) were counted. The number of Nestin^+^/GFAP^+^ cells (C), Ki67^+^ cells (D), and DCX^+^ cells (E) was analyzed by Student's *t* test. The data are presented as the means ± SD. ^#^ *p* < 0.05, ^##^ *p* < 0.01 vs. the BDNF + NC group, ^*^ *p* < 0.05, ^**^ *p* < 0.01, ^***^ *p* < 0.01 vs. the CTR group

To ascertain whether neurotrophins participate in the pathophysiological changes after TBI, we tested the expression of BDNF and NT‐3 in the SVZ (*n* = 3 per group) from 1 to 7 days after surgery (Figure 5C). We found that BDNF expression was upregulated gradually after TBI, while the expression of NT‐3 was not altered. We also tested the expression of DCX, and in accordance with the expression of BDNF, the expression of DCX was found to have increased gradually after TBI. This finding suggested that BDNF participated in SVZ NSC activation. Then, we explored whether miR‐124‐3p directly regulated BDNF and examined the expression of BDNF in the group treated with a miR‐124 agomir or antagomir. The Western blot results showed that the expression of BDNF remained unchanged between each group (Figure 5D). To confirm the role of miR‐124 in BDNF function after TBI, we administered PBS + NC (CTR), BDNF + NC, BDNF + agomir or BDNF + antagomir through lateral intracerebral ventricular injection, and the rats (*n* = 6 per group) were sacrificed 7 days after CCI induction. The IF results showed that BDNF increased the number of GFAP^+^/Nestin^+^ stem cells, DCX^+^ newborn neurons, and Ki67^+^ proliferating cells (Figure 6). These results confirmed that BDNF promoted SVZ NSC activation after TBI. On the contrary, the combined injection of BDNF and antagomir further increased the numbers of GFAP^+^/Nestin^+^ stem cells, DCX^+^ newborn neurons, and Ki67^+^ proliferating cells. Moreover, the BDNF + agomir group had fewer GFAP^+^/Nestin^+^ cells or Ki67+ cells than the BDNF + NC group. These results implied that miR‐124 attenuated the function of BDNF in activating SVZ NSCs post‐TBI.

## 4 DISCUSSION

TBI, a vital cause of death and disability, causes untenable pain and places an unaffordable burden to both patients and their family members.^27^ However, for decades, no drug has been sufficiently effective to alleviate the neurological disabilities caused by TBI. Adult mammalian NSCs, with self‐renewal and differentiation capacity, possess promising potential for neuroregeneration and neuroprotection.^26^ In the adult mammalian brain, NSCs reside in two major neurogenic niches, the SVZ lining the lateral ventricles and the SGZ within the dentate gyrus of the hippocampus. Post‐TBI NSC studies have mostly focused on the SGZ, while the effects of SVZ NSCs and their regulatory patterns have been less extensively explored. In the present study, we confirmed that SVZ NSCs were activated after TBI and that this activation could be regulated by miR‐124‐3p. This regulatory effect was partially realized by blocking downstream pathways of BDNF, including the PI3K/Akt pathway and Ras pathway.

MiR‐124 is one of the most abundant miRNAs in the adult central nervous system (CNS)^28^ and has been reported to regulate SVZ NSC proliferation and differentiation. MiR‐124‐3p has been shown to be downregulated for a long time in the CNS after TBI.^29^ Previous studies on miRNA functions were mostly based on target prediction, and then, the possible impacts of these miRNAs on subjectively selected target were evaluated.^10^ However, considering that one miRNA may regulate many perfectly and imperfectly paired targets, comprehensive understanding of miRNA functions has been difficult to obtain through classical approaches. Hence, we analyzed the predicted targets of miR‐124‐3p with a bioinformatic method and identified the most extensively regulated pathways, including the “neurotrophin signaling pathway,” “prolactin signaling pathway,” and “acute myeloid leukemia.” As miRNA downregulates its targets by targeting the 3’ mRNA UTR with the seed region in the 5’ end of miRNA,^12^ all the pathways that we predicted were thought to be inhibited by miR‐124‐3p.

In the neurotrophin signaling pathway, for which the lowest KEGG pathway enrichment *p*‐value was obtained, the miR‐124‐3p targets were mainly distributed in the downstream TrkB‐Shc‐PI3K‐Akt pathway and TrkB‐SH2B‐Ras pathway. These pathways play important roles in NSC survival, development, and higher‐order activities such as learning and memory. The PI3K/Akt pathway exerts antiapoptotic functions, promoting cell survival, enhancing dendritic growth and branching, and modulating synaptic plasticity. The MAPK/Ras signaling pathway regulates protein synthesis during neuronal differentiation.^30^ Among all the identified targets of miR‐124‐3p, PI3KCA, Akt3, and Ras, which participated in more than eight predicted pathways, were regarded as critical targets, and we verified that they were regulated upon exogenous administration of miR‐124‐3p analogs or inhibitors post‐TBI. In addition, the expression of phospho‐Akt, phospho‐PI3K, phospho‐MEK1/2, and phospho‐Erk1/2 was also affected by miR‐124‐3p interference, which confirmed the regulatory role of this miRNA in the neurotrophin signaling pathway. Considering that BDNF is upregulated after TBI and that miR‐124‐3p attenuates the function of the BDNF pathway, it is reasonable to think that the decrease in miR‐124 expression after TBI confers protection onto the injured brain. Additionally, utilizing a miR‐124 inhibitor to strengthen the effect of BDNF and further improve TBI prognosis is a promising approach. As BDNF is too large to permeate through the blood–brain barrier,^31^ and therefore, direct BDNF injection into the CSF is recommended, as this provides additional beneficial effects to the NSCs along the lateral ventricles.

For the prolactin signaling pathway, for which the second highest score KEGG pathway analysis score was obtained, no method to evaluate its connection with TBI was discerned. Prolactin (PRL) is involved in a wide range of biological functions, including osmoregulation, lactation, reproduction, growth and development, endocrinology and metabolism, brain and behavior, and immunomodulation.^32^ On the basis of the putative targets of miR‐124, we identified 13 targets that were downstream of the prolactin signaling pathway, and these targets participated in the PI3K/Akt, MAPK, and Jak/STAT signaling pathways. PRL functions are not confined to the classic endocrine system; they include promoting neurogenesis, neurodevelopment, neuroplasticity and neuroprotection, broadly affecting memory, cognition, and learning.^33^ Elevated levels of secreted PRL in pregnant and lactating female mice enhanced neurogenesis in the SVZ,^34^ which suggested that miR‐124‐3p downregulation strengthens post‐TBI SVZ NSC activation through the prolactin signaling pathway. This result also offers clues to explain the reason that males and females have different outcomes after TBI.^35^

One of the main limitations in our study is that we only used young adult male rats to assess the role of miR‐124‐3p in TBI prognosis and SVZ stem cell activation. Sex and age play important roles in determining the outcome of brain injury,^36^, ^37^ and premenopausal females often receive a better prognosis than males after brain injury, including TBI.^35^ Individuals of different sexes suffer different pathophysiological changes after brain injury, including discrepant gene expression^38^, ^39^ and immunoreactions.^40^ Therefore, investigation into the effects of miR‐124‐3p on rats of different sexes and ages separately is important, and future clinical trials should consider the impacts of sex and age on TBI prognosis in patient selection and classification.

## 5 CONCLUSION

In summary, our data indicated that downregulation of miR‐124 after TBI enhanced SVZ NSC activation and further improved motor function in adult male rats. These effects were partially realized through intensification of pathway activation downstream of BDNF, such as the PI3K/Akt and Ras signaling pathways. Our exploration also indicated that bioinformatics analyses can lead to a comprehensive view for predicting miRNA functions and may be useful for providing more efficacious guidance for miRNA exploration.

## CONFLICTS OF INTEREST

The authors declare that they have no conflicts of interest.

## Supporting information

Figure S1

Supplementary Material

Supplementary Material

Supplementary Material

Supplementary Material
