# Supplementary figures and images for: Downregulation of microRNA‐124‐3p promotes subventricular zone neural stem cell activation by enhancing the function of BDNF downstream pathways after traumatic brain injury in adult rats
Source: CNS Neurosci Ther. 2022 Apr 28;28(7):1081–92. doi: 10.1111/cns.13845 (PMC9160452; doi:10.1111/cns.13845)

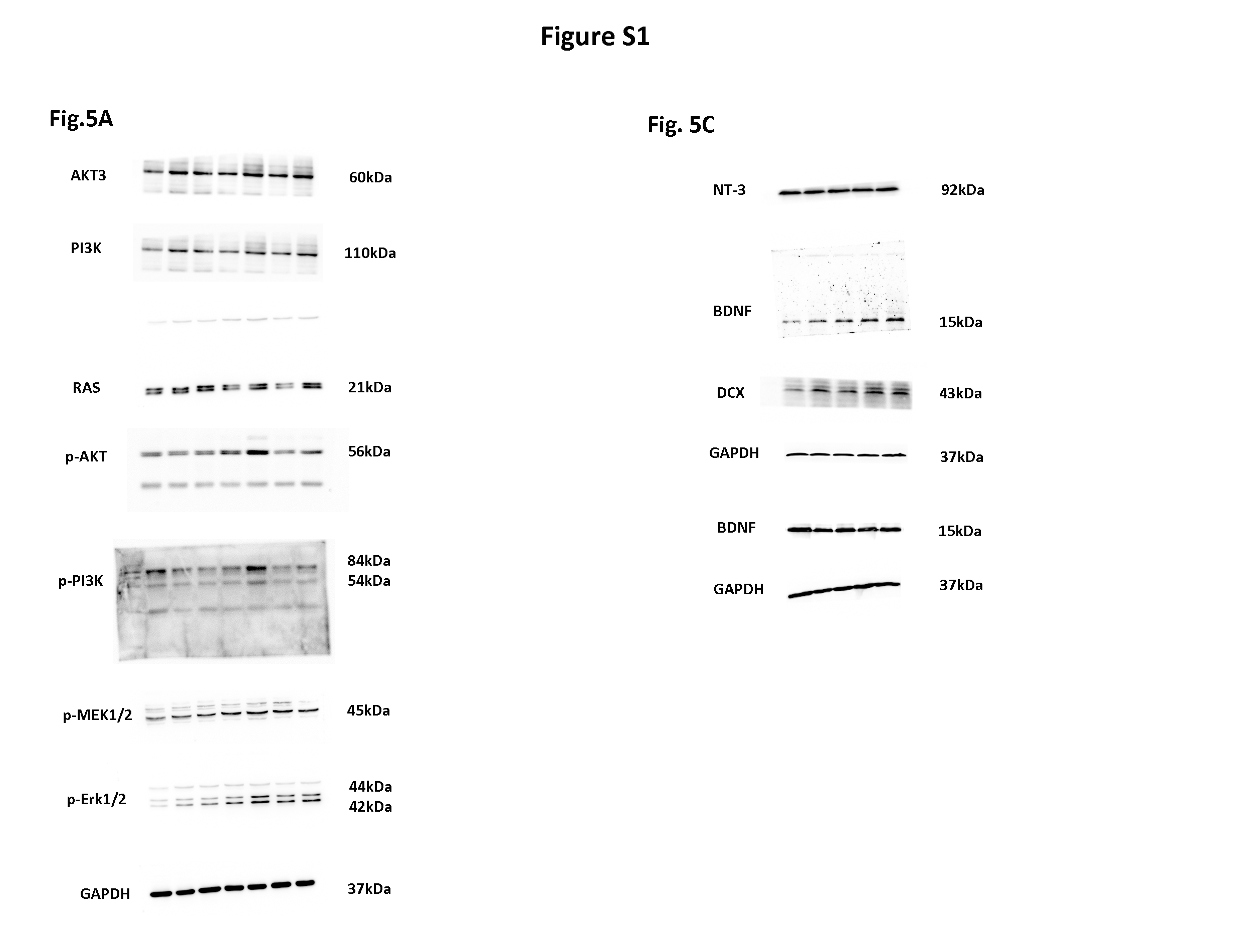

Supplement: Supplementary file 1 — Figure S1 [file CNS-28-1081-s004.tif]

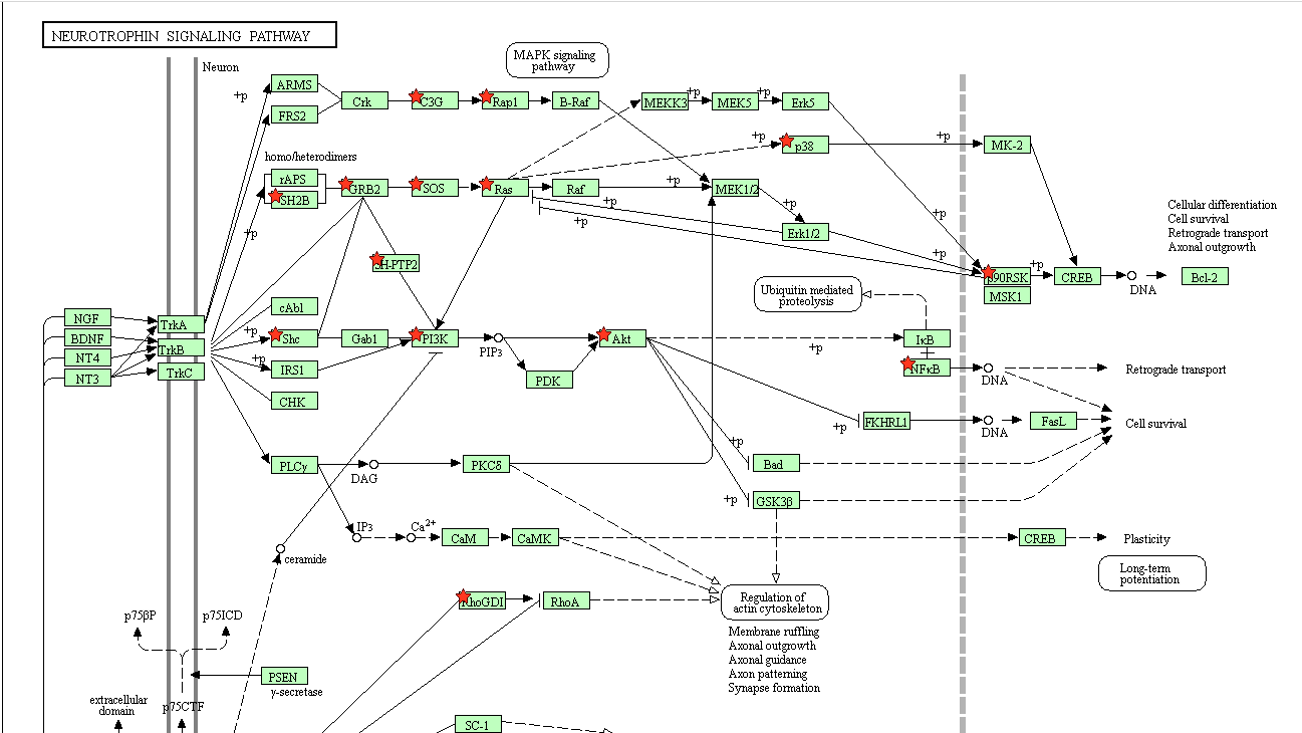

Supplement: Supplementary file 4 — Supplementary Material [file CNS-28-1081-s003.tif]

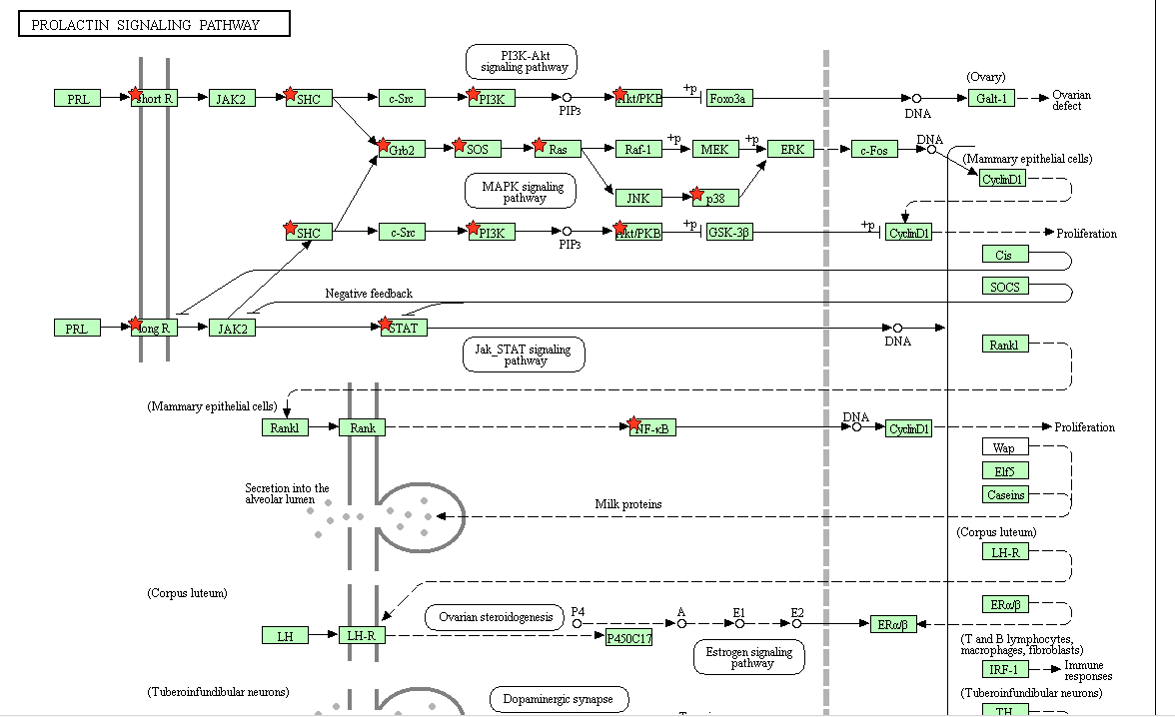

Supplement: Supplementary file 5 — Supplementary Material [file CNS-28-1081-s005.tif]
