# Supplementary material for: Downregulation of microRNA‐124‐3p promotes subventricular zone neural stem cell activation by enhancing the function of BDNF downstream pathways after traumatic brain injury in adult rats
Source: CNS Neurosci Ther. 2022 Apr 28;28(7):1081–92. doi: 10.1111/cns.13845 (PMC9160452; doi:10.1111/cns.13845)

Full unedited gels for figure 5

Akt3

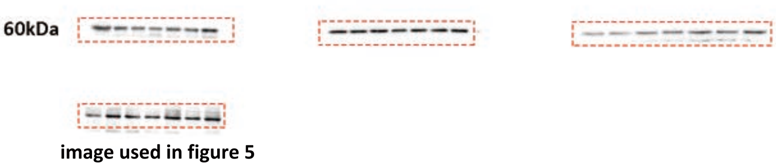

PI3K

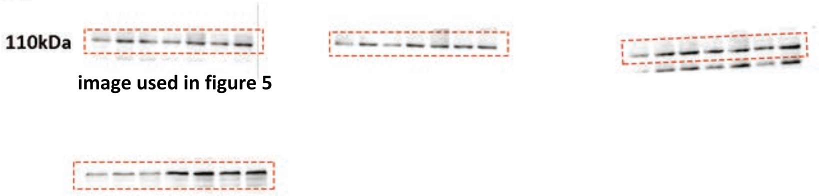

Ras

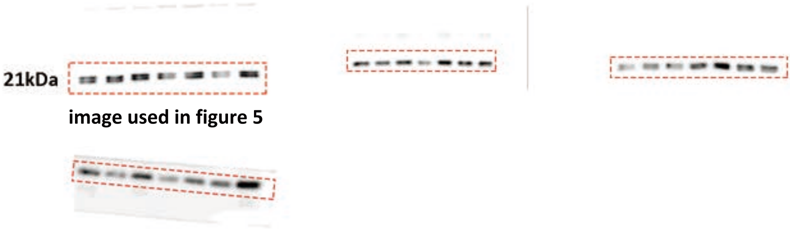

p-Mek

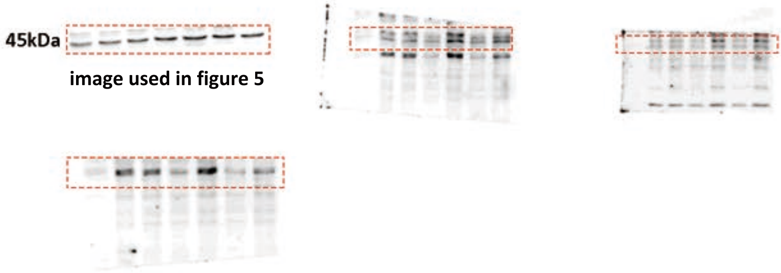

p-Akt

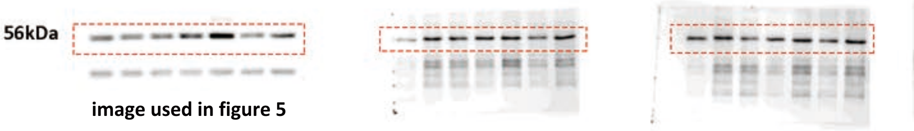

Supplement: Supplementary file 2 — Supplementary Material [file CNS-28-1081-s001.pdf]
