# Supplementary material for: Downregulation of microRNA‐124‐3p promotes subventricular zone neural stem cell activation by enhancing the function of BDNF downstream pathways after traumatic brain injury in adult rats
Source: CNS Neurosci Ther. 2022 Apr 28;28(7):1081–92. doi: 10.1111/cns.13845 (PMC9160452; doi:10.1111/cns.13845)
Supplement: Supplementary file 3 — Supplementary Material [file CNS-28-1081-s002.pdf]

Full unedited gels for figure 5

p-Erk

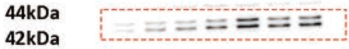

image used in figure 5

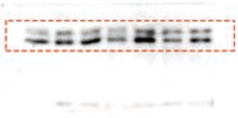

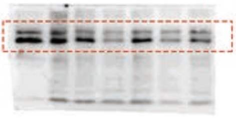

p-PI3K

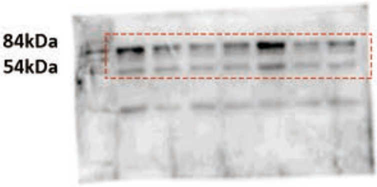

image used in figure 5

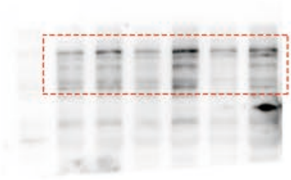

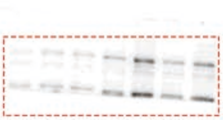

GAPDH

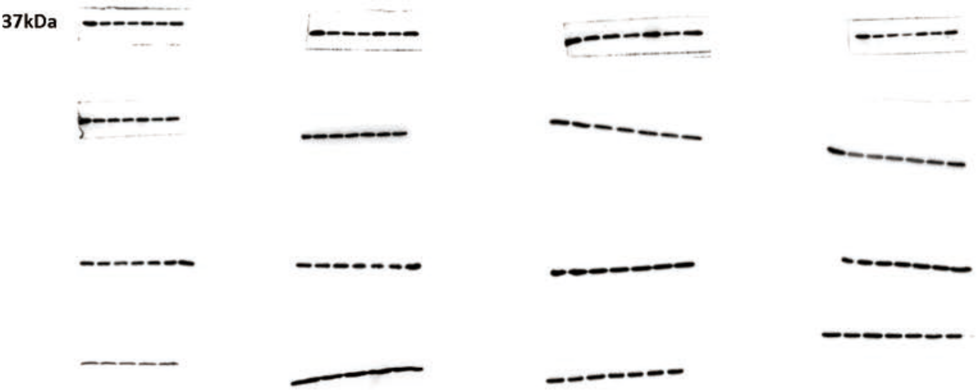

image used in figure 5 image used in figure 5 image used in figure 5

BDNF

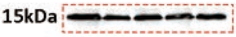

image used in figure 5

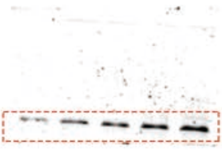

image used in figure 5

DCX

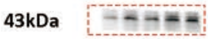

image used in figure 5

NT-3

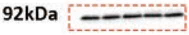

image used in figure 5
